# Socioeconomic Position Early in Adolescence and Mode of Delivery Later in Life: Findings from a Portuguese Birth Cohort

**DOI:** 10.1371/journal.pone.0119517

**Published:** 2015-03-23

**Authors:** Cristina Teixeira, Susana Silva, Milton Severo, Henrique Barros

**Affiliations:** 1 Institute of Public Health, University of Porto, Porto, Portugal; 2 Department of Clinical Epidemiology, Predictive Medicine and Public Health, University of Porto Medical School, Porto, Portugal; 3 Polytechnic Institute of Bragança, Bragança, Portugal; The Ohio State University, UNITED STATES

## Abstract

**Objective:**

This study assessed the influence of socioeconomic position at 12 years of age (SEP-12) on the variability in cesarean rates later in life.

**Methods:**

As part of the Portuguese Generation XXI birth cohort we evaluated 7358 women with a singleton pregnancy who delivered at five Portuguese public hospitals serving the region of Porto (April/2005–September/2006). Based on the twelve items that described socioeconomic circumstances at age 12, a latent class analysis was used to classify women’s SEP-12 as high, intermediate and low. Multiple Poisson regression was used to estimate adjusted risk ratio (RR) and respective 95% confidence interval (95% CI).

**Results:**

The cesarean rates in high, intermediate and low SEP-12 were, respectively, 40.9%, 37.5% and 40.5% (p = 0.100) among primiparous women; 14.2%, 11.6% and 15.5% (p = 0.04) among multiparous women with no previous cesarean and 78.6%, 72.2% and 70.0% (p = 0.08) among women with a previous cesarean. A low to moderate association between SEP-12 and cesarean rates was observed among multiparous women with a previous cesarean, illustrating that women from higher SEP-12 were more likely to have a surgical delivery (RR = 1.12;95%CI:1.01–1.24 comparing high with low SEP-12 and RR = 1.03:95%CI:0.94–1.14 comparing intermediate with low SEP-12) not explained by potential mediating factors. No such association was found either in primiparous or in multiparous women without a previous cesarean.

**Conclusions:**

The association between SEP-12 and cesarean rates suggests the effect of past socioeconomic context on the decision concerning the mode of delivery, but only among women who experienced a previous cesarean. Accordingly, it appears that early-life socioeconomic circumstances drive cesarean rates but the effect can be modified by lived experiences concerning childbirth.

## Introduction

Europe has witnessed a marked increase in the frequency of cesarean delivery during the last three decades [1] with 80% of the countries presenting rates that exceed the upper limit of 15% proposed by the World Health Organization (WHO). [2] This is a matter of concern because cesarean section has been associated with higher risk of severe maternal morbidity including thromboembolism, [3] puerperal infection, [4, 5] hysterectomy, [3, 5] as well as abnormal placentation in subsequent pregnancies. [5] The risk of major complications increases with the increasing number of previous cesarean deliveries. [5] Unnecessary cesarean result in avoidable suffering and wasted health care resources from unduly allocated staff, surgery procedures and longer hospital stays. [6] Addressing this apparent epidemic of cesarean in countries such as Portugal where cesarean rate was 36% in 2011, [7] is not only a challenge for obstetric care professionals but also a broader public health issue.

Medical technologies coupled with an increase in obstetric interventions that influence the time and the mode of birth, have been faced as the norm in childbirth and partly explain the current trends of cesarean rates in Europe. [8, 9] Within this context of medicalized birth, parents and caregivers can negotiate, formally or informally, the decision to perform a cesarean section. [10–12] In this process, ambiguities in diagnosis [13, 14] and the practice of defensive medicine [15] allow non-clinical factors to become key elements in the decision-making process. At the basis of such a decision is the encounter between the woman and her caregiver, in which woman’s preferences and views regarding childbirth [16–19] and the clinician’s subjective interpretation of her obstetric risks and the perception of desired mode of delivery [10, 15] are likely to influence the decision to proceed with cesarean.

Parents’ views on the safest and most fulfilling mode of delivery are shaped by embedded social relations; giving birth is an embodied experience where individual choices and actions are influenced by the social structure. [20] A large body of research has shown a clear association between cesarean rates and socioeconomic factors [11, 18, 21–27] apart from the woman´s obstetric risk, [11, 21, 25, 26] even where access to health care services is not an issue. [11, 22, 26, 27] These findings suggest that woman’s preference or her ability to engage in the decision-making process concerning mode of delivery, are likely shaped by her socioeconomic context, which drives cesarean rates. Studies reporting this sort of influence on the mode of delivery have mainly focused on women’s socioeconomic position at the moment of delivery. [11, 18, 21–28]

Growing evidence suggests that early socioeconomic context shapes the decision-making process about health related choices, influencing health behaviors such as alcohol consumption, [29–32] smoking, [31, 33–35] physical activity, [29, 30, 34, 36] and dietary intake. [29, 34] According to a life course perspective there are sensitive periods when individuals are more vulnerable to the socioeconomic factors [37, 38] and adolescence has been considered a crucial time period in determining health behaviors and attitudes. [37]

However, to the best of our knowledge, there is no published data on the influence of early socioeconomic circumstances on cesarean rates. While early and current socioeconomic positions are correlated, [29, 39] it seems important to disentangle such influences in order to understand the factors that drive the decision-making process concerning the mode of delivery.

To gain a deeper understanding of the effect of socioeconomic factors on cesarean rates, we evaluated the influence of women’s socioeconomic position at the beginning of adolescence (12 years of age) on having a cesarean delivery later in life.

## Materials and Methods

In Portugal, where nearly all deliveries occur within hospitals, the National Health System provides antenatal, obstetric and neonatal care funded by public resources free of charge, at the point of use, for all childbearing women and their babies. There is also a market supply of private health care services. Although almost 40% of Portuguese pregnant women choose to have prenatal care under private physicians, 90% of them deliver in public hospitals. [7] The majority of these deliveries take place in obstetric units that offer the highest level of obstetric and neonatal care (level III). In the Portuguese National Health Service, cesarean is a medical decision based on guidelines in which maternal request is not recognized as an acceptable indication for surgical delivery.

### Study sample

The participants were recruited during the assembling of a birth cohort (Geração XXI), in Porto, in the North of Portugal. From April 2005 to August 2006, women delivered at the five public maternity units in the Porto Metropolitan Area were invited to participate. After the refusal of 8% of those invited, the final sample comprised 8495 women who delivered live born infants (> 24 weeks) and 8351 of these women delivered a singleton baby. Individual interviews performed 24 to 72 hours after delivery by trained interviewers provided information on demographic characteristics past and current socioeconomic circumstances, obstetric and gynecological history, lifestyles and current pregnancy events using a structured questionnaire. Information on pregnancy complications, delivery circumstances and data on newborn characteristics were abstracted from medical records.

The study was approved by the Ethics Committee of the University of Porto Medical School/Hospital S. João and written informed consent was obtained from each participant. Informed consent on behalf of the children was obtained from the mothers in writing. Data were anonymously analyzed both regarding the individuals and the hospitals where delivery took place.

For the purpose of the present research we excluded twin pregnancies (n = 144), women living with neither their mother nor their father at 12 years old (n = 617), and immigrant women (n = 322) as classified elsewhere [40] or those with no information about migration status (n = 51). Briefly, a woman was classified as immigrant if she was born abroad and both parents were foreign-born or if one or both parents were Portuguese-born but woman moved to Portugal at the age of 18 or later. Of the 7361 eligible mothers, 7358 (99.9%) had information allowing their classification according to the socioeconomic circumstances when they were 12 years old.

### Socioeconomic position at 12 years old

Based on retrospective recall at the time of delivery, we obtained information on twelve items that we used to describe the socioeconomic position when the participants were 12 years of age (SEP-12). Nine items asked about family assets: house ownership, heating system at home, washing machine, television, telephone, housemaid, bicycle, car ownership, and spending holidays away from home at least one week per year. Two items asked whether at 12 years old the participants were members of a club or an association (cultural, sports or other). The final item considered the number of years of schooling of the father (or of the mother if living only with her) dichotomized using completed 4 years as the cutoff point. The response to each of these items was considered as yes/no options and the information was used to develop a latent class model in order to create distinct categories of social position. The questions on such items are provided as supporting information (S1 Text).

### Outcome

The study outcome was the mode of delivery, categorized as vaginal or cesarean section. This information was obtained from medical records.

### Covariates

The following variables were considered possible mediating factors in the association between SEP-12 and the mode of delivery: maternal age, past obstetric history (classified as primiparous and multiparous with or without a previous cesarean), completed years of schooling (<9, 9–12 and >12), current household income per month (= <1000; 1001–1500 and > = 1500 euros), body mass index (<25.0, 25.0–29.9 and > = 30 Kg/m^2^), chronic diseases (e.g. hypertension, diabetes, asthma), type of antenatal care (only public services or at most one visit to a private doctor or service), pregnancy complications (placental disorders, gestational diabetes, pregnancy induced hypertensive disorders), the duration of pregnancy (<37, 37–40 and >40 weeks), fetal presentation (cephalic or non-cephalic), the hospital where delivery took place (numbered from 1 to 5) and sex-specific birth weight for gestational age (<10^th^ percentile—small, > = 90^th^ percentile—large, and otherwise—adequate) according to a population-based reference. [41] We considered the covariates as possible mediating factors rather than confounding factors because we can hipothetize plausible causal pathways between SEP-12 and the mode of delivery by including all the covariates.

### Statistical analysis

Women’s SEP-12 was obtained using latent class analysis (LCA) based on the twelve items earlier described. The LCA considered that the performance of each participant in this set of items was explained by a categorical latent variable with K classes, commonly called “latent classes”. This approach allowed us to identify the optimal number of groups that capture heterogeneity across the participants in their responses on such items, uncovering distinct groups of individuals from the sample, homogeneous within the group. In this study, the number of latent classes was defined according to the Bayesian information criterion (BIC). We started the analysis from one single class and we increased one class at each step. The BIC values decreased as the number of classes increased and the best solution was identified when BIC values leveled-off. Interpretation of the model was based on item profiles in each category, obtained from the probabilities of endorsing each item response, conditional on class membership. [42, 43]

We tested several models, specifying between one and five latent classes. The BIC values decreased with increasing class numbers but leveled off between three and four (Table 1). The three levels solution provided a more coherent interpretation of SEP-12. According to the latent class model (Table 2), a decreasing prevalence of assets and indicators of wellbeing was found as women were categorized as higher (class 1), intermediate (class 2) and lower (class 3) social position at 12 years of age. LCA models were fitted using MPlus (version 5.2; Muthen & Muthen).

**Table 1:** Latent class analysis for socioeconomic circumstances at 12 years of age.

Number of classes	Log likelihood	Number of parameters	BIC*
1	−50346.696	12	100801.496
2	−45338.813	25	90902.843
**3**	−**44293.921**	**38**	**88930.173**
4	−44116.760	51	88692.964
5	−43942.707	64	88461.971

*BIC—Bayesian information criterion

**Table 2:** Marginal percentage of subjects with positive answer to the item in each assigned class of the 3-classes latent class model.

	Total	class 1	class 2	class 3
item	7358 (100.0)	1752 (24.0)	3806 (52.0)	1800 (24.0)
house ownership	47.4	69.7	41.2	38.3
car ownership	54.2	95.5	55.2	14.2
television	97.0	99.7	100	88.5
bicycle	75.4	96.7	81.0	45.0
housemaid	10.7	40.7	1.4	1.0
spending holydays away home	38.8	90.6	30.9	6.4
telephone	70.5	98.1	84.6	18.0
washing machine	70.4	95.6	87.7	13.9
heating system	76.9	97.6	86.2	40.1
member of an association	27.9	41.4	25.4	20.2
member of a club	21.1	42.1	17.9	7.9
father’s years of schooling > 4	27.2	65.0	18.6	6.0

Given 32% of women included in the LCA presented a missing value in at least one of the twelve items used to characterize past socioeconomic circumstances, we used full information maximum likelihood estimation to handle missing values, assuming missing at random. [44] The proportion of missing values in each of twelve items varied between less than 1% and 20%. Sensitivity analyses allowed us to evaluate the effect of missing data on LCA by excluding from the LCA the items with more than 15% of missing data (heating system at home, television and father’s years of schooling > 4). The agreement between a model with 12 items and the model with only 9 items was analyzed by using Cohen´s Kappa Statistics, which value (kappa = 0.85) provides some assurance against substantial bias in the LCA. [45]

Multiple Poisson regression with robust variance was used to estimate adjusted risk ratio (RR) and respective 95% confidence interval (95% CI). Given cesarean is a common event in our setting (35% of all deliveries), this approach was preferred to logistic regression in order to avoid overestimation of measures of association. [46]

Poisson regression analyses were stratified by the three categories of past obstetric history (primiparous, multiparous with no previous cesarean and multiparous with previous cesarean). A basic model with SEP-12 was fitted on mode of delivery and covariates were introduced in the model based on a conceptual framework with three levels of determination defined according to temporal relationships between variables. [47] The first level included maternal characteristics that could be identified at beginning of pregnancy: maternal age, chronic diseases, maternal body mass index, and current socioeconomic indicators (education level and household income per month). The second level incorporated variables associated with pregnancy (type of antenatal care and pregnancy complications). The third level included characteristics at delivery (gestational age, fetal presentation, sex-specific birth weight for gestational age and hospital where delivery took place). From the basic model, multiple regression analyses were conducted by adding covariates according to the following order of variable entry: (1) covariates in the first level of determination; (2) covariates in the second level of determination and (3) covariates in the third level of determination. At each level of determination, covariates were checked one by one and were included in final models if they changed the RR estimates at least by 10.0%. Variance inflation factors test and tolerance values were used to check co-linearity between independent variables. Analyses restricted to women who are mostly close in age (25–34 years old) and restricted to multiparous women with only a previous delivery were also performed. In all Poisson regression models each categorical variable was introduced in the model with an additional category for missing values Statistical analysis was performed with IBM SPSS Statistics (version 19.0) and R (version 2.13.0). The level of significance was set at p<0.05. Dataset is provided as Supporting Information (S1 Dataset).

## Results

As shown in Table 3, the distribution of actual socioeconomic, demographical, clinical and health care characteristics was significantly different according to the SEP-12. Women with a less favorable SEP-12 were older, more frequently multiparous and obese and presented higher proportion of pregnancy complications.

**Table 3:** Sample characteristics according to the SEP-12.

	Total	According to the Socioeconomic Position at 12 years old			
		High	Intemediate	Low	
	n = 7358	n = 1752	n = 3806	n = 1800	p-value*
	n (%) or mean ± standard deviation				
**Maternal age (years)**	29.5±5.59	29.9±5.06	28.4±5.48	31.4±5.73	<0.001
**Current education level (years)**					
<9	2200 (29.9)	84 (4.8)	1043 (27.3)	1073 (59.6)	<0.001
9–12	3512 (47.7)	769 (43.9)	2141 (56.3)	602 (33.5)	
>12	1638 (22.3)	896 (51.1)	619 (16.3)	123 (6.8)	
missing	8 (0.1)	3 (0.2)	3 (0.1)	2 (0.1)	
**Current household income per month (euros)**					
= <1000	2511 (34.1)	276 (15.8)	1369 (36.0)	866 (48.1)	<0.001
1001–1500	1875 (25.5)	388 (22.1)	1061 (27.9)	426 (23.7)	
>1500	2003 (27.2)	865 (49.4)	890 (23.4)	248 (13.8)	
Did not know/did not want to report	969 (13.2)	223 (12.7)	486 (12.8)	260 (14.4)	
**Past obstetric history**					
primiparous	4111 (50.9)	1108 (63.2)	2295 (60.3)	708 (39.3)	<0.001
multiparous no previous cesarean	2307 (31.4)	415 (23.7)	1080 (28.4)	812 (45.1)	
multiparous no previous cesarean	940 (12.8)	229 (13.1)	431 (11.3)	280 (15.6)	
**Body Mass Index (Kg/m** ^**2**^)					
<25	4624 (62.8)	1255 (71.6)	2373 (62.3)	996 (55.3)	<0.001
25–29	1487 (20.2)	260 (14.8)	784 (20.6)	443 (24.6)	
> = 30	612 (8.4)	86 (4.9)	310 (8.1)	216 (12.0)	
missing	635 (8.6)	151 (8.6)	339 (8.9)	145 (8.1)	
**Chronic pre-pregnancy disease**					
Yes	930 (12.6)	227 (13.0)	463 (12.2)	240 (13.3)	0.443
No	6416 (87.2)	1523 (86.9)	3333 (87.6)	1560 (86.7)	
missing	12 (0.2)	2 (0.1)	10 (0.3)	0 (0.0)	
**Antenatal care**					
Only public services	4309 (58.6)	676 (38.6)	2358 (62.0)	1275 (70.8)	<0.001
At least 1 visit at private services	2780 (37.8)	1034 (59.0)	1288 (33.8)	458 (25.4)	
missing	269 (3.7)	42 (2.4)	160 (4.2)	67 (3.7)	
**Pregnancy complications**					
Yes	843 (11.5)	168 (9.5)	420 (11.0)	255 (14.2)	<0.001
No	6483 (88.1)	1576 (90.0)	3372 (88.6)	1535 (85.2)	
missing	32 (0.4)	8 (0.5)	14 (0.4)	10 (0.6)	
**Gestational age**					
<37	551 (7.5)	144 (8.2)	262 (6.9)	145 (8.1)	0.271
37–40	6321 (85.9)	1502 (85.7)	3288 (86.4)	1531 (85.0)	
>40	480 (6.5)	105 (6.0)	253 (6.6)	122 (6.8)	
missing	6 (0.1)	1 (0.1)	3 (0.1)	2 (0.1)	
**Fetal Presentation**					
cephalic	6833 (92.9)	1669 (93.0)	3534 (92.9)	1630 (92.7)	0.971
non-cephalic	414 (5.6)	99 (5.7)	216 (5.7)	99 (5.5)	
missing	111 (1.5)	23 (1.3)	56 (1.5)	32 (1.8)	
**Sex-specific birthweight for gestational age**					
normal	5966 (81.2)	1424 (81.3)	3097 (81.4)	1445 (80.3)	0.761
Small (<10th percentile)	1094 (14.9)	261 (14.9)	564 (14.8)	269 (14.9)	
Large (> = 90th percentile)	277 (3.6)	63 (3.6)	137 (3.6)	77 (4.3)	
**Hospital where delivery took place**					
**1**	1734 (23.6)	324 (18.5)	931 (24.5)	479 (26.6)	<0.001
**2**	1268 (17.2)	354 (20.2)	599 (15.7)	315 (17.5)	
**3**	789 (10.7)	248 (14.2)	336 (8.8)	205 (11.4)	
**4**	1793 (24.4)	497 (28.4)	894 (23.5)	402 (22.3)	
**5**	1774 (24.1)	329 (18.7)	1046 (27.5)	399 (22.2)	
**Mode of delivery**					
vaginal	4761 (64.7)	1060 (60.5)	2510 (65.9)	1191 (66.2)	<0.001
caesarean section	2597(35.3)	692 (39.5)	1286 (34.1)	609 (33.8)	

* p-value for chi-square test; missing values were excluded from the computation

The prevalence of cesarean section was 38.9% in primiparous (40.9% in the high, 37.5% in the intermediate and 40.5% in the low SEP-12 category; p = 0.100), 13.4% in multiparous without previous cesarean (14.2% in the high, 11.6% in the intermediate and 15.5% in the low SEP-12 category; p = 0.04) and 73.1% in multiparous with previous cesarean (78.6% in the high, 72.2% in the intermediate and 70.0% in the low SEP-12 category; p = 0.08).

Poisson regression analyses to explore the association between SEP-12 and the cesarean rates were conducted separately by past obstetric history, using low SEP-12 as the reference group (Table 4). The basic model reveals there were no differences in the risk of cesarean section according to the SEP-12 among primiparous women. Among multiparous with no previous cesarean section, those belonging to the intermediate SEP-12 group were less likely to have a cesarean when compared with women from the low SEP-12 group (RR = 0.75: 95%CI: 0.59–0.94), but no differences were observed for women from the high group in comparison with women from the low SEP-12 group. Instead, a gradient according to the SEP-12 was seen for cesarean rates among multiparous women with previous cesarean, so that women from high SEP-12 were more likely to have a surgical delivery. Considering low SEP-12 as the reference group, the RR estimates were 1.12 (95%CI: 1.01–1.24) and 1.03 (95%CI: 0.94–1.14) for women belonging to high SEP-12 and intermediate SEP-12, respectively.

**Table 4:** Risk ratio for cesarean delivery by SEP-12.

		Primiparous	Multiparous no previous cesarean	Multiparous with previous cesarean
		n = 4111	n = 2307	n = 940
Variables included in the models at each stage	SEP-12	RR (95% CI)		
**basic model**	**Low**	1	1	1
only SEP-12	**Intermediate**	0.92 (0.83–1.03)	0.75 (0.59–0.94)	1.03 (0.94–1.14)
	**High**	1.01 (0.90–1.13)	0.92 (0.69–1.22)	1.12 (1.01–1.24)
**basic model + variables considered in the first level of determination**	**Low**	1	1	1
SEP-12 + maternal age+ body mass index + current maternal education	**Intermediate**	1.03 (0.93–1.15)	0.82 (0.65–1.05)	1.07 (0.96–1.18)
+ current household income per month + pre-pregnancy chronic diseases	**High**	1.09 (0.97–1.24)	0.98 (0.70–1.35)	1.13 (0.99–1.28)
**basic model + variables considered in the second level of determination**	**Low**	1	1	1
SEP-12+type of antenatal care + pregnancy complications	**Intermediate**	0.93 (0.84–1.03)	0.76 (0.60–0.96)	1.02 (0.92–1.12)
	**High**	0.98 (0.87–1.10)	0.93 (0.68–1.27)	1.09 (0.98–1.22)
**basic model + variables considered in the third level of determination**	**Low**	1	1	1
SEP-12+gestational age at delivery + birth weight for gestational age	**Intermediate**	0.92 (0.83–1.01)	0.77 (0.63–0.96)	1.03 (0.93–1.13)
+ fetal presentation + hospital where delivery took place	**High**	1.02 (0.92–1.13)	0.99 (0.76–1.30)	1.14 (1.03–1.26)
**Final Model**	**Low**	1		1
**Only SEP-12**	**Intermediate**	0.92 (0.83–1.03)		1.03 (0.94–1.14)
	**High**	1.01 (0.90–1.13)		1.12 (1.01–1.24)
**Final Model**	**Low**		1	
**SEP-12 + maternal age + current household income per month**	**Intermediate**		0.81 (0.64–1.03)	
	**High**		0.92 (0.67–1.25)	

Covariates were included in the models according to levels of determination previously defined. Within each level of determination RR estimates were obtained when all covariates were considered in the model (Table 4). Then variables were checked one by one in order to know whether they should keep in the final model. Among primiparous women none of the covariates changed RR estimates at least by 10%. Maternal age and current household income per month explained differences in cesarean section according to the SEP-12 among multiparous women without previous cesarean and such variables were included in the final model. Instead, the gradient according to the SEP-12 for cesarean rates remained among multiparous women with previous cesarean. In this group of women, covariates in the model did not substantially change the RR estimates, whichever the level of determination. Variables included in final models and respective RR estimates for cesarean section by SEP-12 are also presented in Table 4.

Analyses restrict to women aged between 25 and 34 years old (Table 5) and restricted to multiparous women with only a previous delivery (Table 6) revealed similar results to those obtained from analyses applied to the whole sample.

**Table 5:** Risk ratio for cesarean delivery by SEP-12 among women aged between 25 and 34 years old (n = 4545).

		Primiparous	Multiparous no previous cesarean	Multiparous with previous cesarean
		n = 2647	n = 1369	n = 529
Variables included in the models at each stage	SEP-12	RR (95% CI)		
**basic model**	**Low**	1	1	1
only SEP-12	**Intermediate**	0.98 (0.87–1.11)	0.89 (0.65–1.22)	1.07 (0.92–1.23)
	**High**	0.99 (0.86–1.13)	1.02 (0.69–1.51)	1.17 (1.01–1.37)
**basic model + variables considered in the first level of determination**	**Low**	1	1	1
SEP-12 + maternal age+ body mass index + current maternal education	**Intermediate**	1.04 (0.91–1.19)	0.88 (0.64–1.21)	1.10 (0.94–1.28)
+ current household income per month + pre-pregnancy chronic diseases	**High**	1.06 (0.91–1.23)	0.99 (0.63–1.54)	1.18 (0.98–1.43)
**basic model + variables considered in the second level of determination**	**Low**	1	1	1
SEP-12+type of antenatal care + pregnancy complications	**Intermediate**	0.98 (0.87–1.12)	0.90 (0.66–1.23)	1.05 (0.91–1.22)
	**High**	0.98 (0.85–1.13)	1.03 (0.68–1.57)	1.16 (0.99–1.36)
**basic model + variables considered in the third level of determination**	**Low**	1	1	1
SEP-12+gestational age at delivery + birth weight for gestational age	**Intermediate**	0.95 (0.85–1.07)	0.87 (0.65–1.15)	1.06 (0.92–1.22)
+ fetal presentation + hospital where delivery took place	**High**	1.00 (0.88–1.14)	1.07 (0.74–1.54)	1.19 (1.02–1.39)
**Final Model**	**Low**	1		1
**Only SEP-12**	**Intermediate**	0.98 (0.87–1.11)		1.07 (0.92–1.23)
	**High**	0.99 (0.86–1.13)		1.17 (1.01–1.37)
**Final Model**	**Low**		1	
**SEP-12 + maternal age + current household income per month**	**Intermediate**		0.89 (0.65–1.22)	
	**High**		1.02 (0.69–1.51)	

**Table 6:** Risk ratio for cesarean delivery by SEP-12 among multiparous women with only a previous delivery (n = 2403).

		Multiparous no previous cesarean	Multiparous with previous cesarean
		n = 1670	n = 733
Variables included in the models at each stage	SEP-12	RR (95% CI)	
**basic model**	**Low**	1	1
only SEP-12	**Intermediate**	0.80 (0.60–1.05)	1.05 (0.94–1.17)
	**High**	0.90 (0.64–1.27)	1.12 (0.99–1.26)
**basic model + variables considered in the first level of determination**	**Low**	1	1
SEP-12 + maternal age+ body mass index + current maternal education	**Intermediate**	0.91 (0.69–1.21)	1.10 (0.98–1.24)
+ current household income per month + pre-pregnancy chronic diseases	**High**	1.09 (0.75–1.60)	1.17 (1.00–1.36)
**basic model + variables considered in the second level of determination**	**Low**	1	1
SEP-12+type of antenatal care + pregnancy complications	**Intermediate**	0.81 (0.61–1.07)	1.04 (0.93–1.16)
	**High**	0.95 (0.66–1.37)	1.11 (0.98–1.26)
**basic model + variables considered in the third level of determination**	**Low**	1	1
SEP-12+gestational age at delivery + birth weight for gestational age	**Intermediate**	0.79 (0.61–1.02)	1.03 (0.93–1.15)
+ fetal presentation + hospital where delivery took place	**High**	0.97 (0.70–1.34)	1.13 (1.00–1.28)
**Final Model:**	**Low**	1	1
**Only SEP-12**	**Intermediate**	0.80 (0.60–1.05)	1.05 (0.94–1.17)
	**High**	0.90 (0.64–1.27)	1.12 (0.99–1.26)

## Discussion

We sought to examine the association between socioeconomic position at 12 years of age and the probability of cesarean section later in life. Our results provide evidence that women with a previous cesarean section constitute a particular group who showed a gradient according to the SEP-12 for cesarean rates revealing that women from more socioeconomically advantaged families were more likely to repeat a cesarean section. In contrast, socioeconomic circumstances at beginning of adolescence had no significant effect on the probability of cesarean section among primiparous or multiparous women with no previous cesarean section.

The past obstetric history seems to have an effect on women´s preferences regarding the mode of delivery; according to a recent systematic review, women who experienced a cesarean section are more likely to prefer a surgical delivery than women without previous cesarean section. [48] An unpleasant experience from a previous complicated birth leading to an emergency cesarean partially explains such preferences. [19, 49, 50] However, even women who experienced a previous elective surgical delivery are also more likely to prefer a repeat cesarean. [17, 19] While previous studies reported the preference for surgical delivery in women who experienced a previous cesarean, our findings add that variability in prevalence of repeat cesarean delivery depends on women’s socioeconomic position at beginning of adolescence.

Decisions concerning the mode of delivery are the result of a complex interplay between the woman and her caregiver, [51] partly because the best mode of delivery is not always clear-cut. The main clinical indications to proceed with a cesarean section are fetal distress and dystocia. However, the diagnosis of such obstetric conditions entails varying degrees of ambiguity which preclude the appropriateness of obstetric intervention. [13, 14] In this medical context, the decision concerning the mode of delivery could be influenced by other factors rather than obstetric conditions. [10, 12] Such non-medical factors include the woman’s views, knowledge and skills coupled with her experiences regarding childbirth [12, 17, 19] which interact with the provider´s subjective interpretation of obstetric risk, [10, 12] the perception of a woman´s desired mode of delivery and the fear of litigation when risks and benefits of surgical delivery are under discussion. [15]

Women’s influence on the mode of delivery has been associated with socioeconomic factors. Although in some settings, higher levels of maternal education are associated with lower rates of surgical delivery, [11, 21, 24, 26] in other settings higher cesarean section rates were observed among wealthier and more educated women. [22, 23, 25] All these studies addressed only the latest socioeconomic position achieved by the pregnant women. However, a body of research has suggested that, not only adulthood socioeconomic position, but also early socioeconomic circumstances are likely to shape health-related choices and behaviors in adulthood, which impact on rates of health outcomes. [29–36] Our study focused specifically on the contribution of women’s early socioeconomic position to the prevalence of cesarean delivery.

A life-course approach to health recognizes that the environmental context at each life stage reproduces a chain of risk and expectations that impact on health status, through a set of potentially additive pathways. [37, 38] According to this perspective, health-related choices and behaviors are shaped by socioeconomic circumstances from childhood to adulthood, which determine variability in rates of health outcomes at adult age. [29–36] At the same time, other factors such as the embodied lived experiences, intersect with individual trajectories at each life stage, creating new repertoires of perceptions and expectations concerning the management of health risks. [29, 38, 52, 53] This means that socioeconomic context throughout life course has an effect on health-related choices and behaviors. However, this effect is not irreversible and it can be modified by the interplay of other circumstances that provide constraints or encouragement for the expression of a particular health-related behavior. [29, 38] Our findings fit in this framework. Firstly, we found variability in cesarean rates by SEP-12, which suggests the effect of socioeconomic context at the beginning of adolescence in shaping a woman´s behavior towards childbirth. Secondly, according to our results such an effect was modified by different experiences concerning childbirth; the effect of early socioeconomic context had no expression among women with no experience of a surgical delivery but was evident among women with a previous cesarean section.

Variability in rates of elective and emergency cesarean section has been associated with women´s preferences for cesarean section. [17] However, women with higher education levels are more likely to have preferences for a cesarean section fulfilled probably because they have more skills to engage in the decision-making process. [17] If women who experienced a cesarean section are more likely to prefer a surgical delivery, [48] variability in cesarean rates by SEP-12 we found in this group of women could be explained by differential skills to interact with care providers.

A major strength of this study is the large set of data on sociodemographic characteristics, gynecological history and current pregnancy events, which allowed us to check multiple potential mediating factors for the association between cesarean section and the exposure of interest. Furthermore, we used a comprehensive conceptual framework in which potential mediating factors were considered according to three levels of determination [47] to understand whether the association between SEP-12 and cesarean section could be explained by factors identified chronologically at begin of pregnancy, during pregnancy and at delivery.

Some consideration must be given to the limitations of this study. Data collection on a large set of variables is usually based on long questionnaires that are tiring to fill out and so missing data are almost unavoidable. [44] Indeed, in our study one third of the women included in LCA had no information on at least one of the twelve items used to characterize past socioeconomic circumstances. However, less than 3% of women had missing values on more than three items. The LCA allowed us to classify women based on a multidimensional set of items providing a detailed description of what defines more or less advantageous socioeconomic circumstances when women were 12 years old. However, information on such items was based on recall at time of delivery that would likely have resulted in exposure misclassification. Furthermore, questionnaires used to collect the data were not validated in the Portuguese context. Still, in accordance with other researchers, [43] we recognized that LCA results depend on the items entered into the latent class model. Thus, the classification of women according to the socioeconomic circumstances at the beginning of adolescence was a crucial issue.

Socioeconomic position is a complex attribute encompassing material and non-material resources, both crucial concerning health-related choices. [39, 54] Asset-based indicators have been commonly used to assess material resources, known as economic capital. [54] Non-material resources encompass informational resources, knowledge and skills, usually referred to as cultural capital, as well as a set of relationships with networks and other social structures, called social capital. [55] The dominant measures of cultural capital used for epidemiological studies in high-income countries focus on institutionalized knowledge and skills (years of formal education completed or qualifications attained). [39] Social capital has been assessed through membership of community organizations (hobby or sports groups, or civic or social organizations). [43, 56] In this study we intended to characterize socioeconomic circumstances by using material and non-material indicators when women were aged 12 years. We considered the information on ownership of a range of household amenities as an attempt to measure early-life material living conditions. We also included membership of the participants, when they were 12 years of age, of a club or association as a proxy of family´s relationship with the community. The cultural capital evaluation of women’s parents included the completion of elementary school, seen as an attempt to capture knowledge-based assets of women´s parents. For people born before 1966 in Portugal (99% of women’s parents were born before 1966), elementary school corresponds to 4 years of schooling and few people had access to high school. This is the reason why we used 4 years as cut-off to classify women’s parents according to the formal education completed.

The asset-based indicators that we use in this study to characterize past socioeconomic position could have a different meaning across countries, particularly if there are large differences in socioeconomic indicators between them. This is the reason why we excluded immigrant women from our analyses.

We thought that the asset-based indicators could also have a different meaning across years, given the natural development of Portuguese society. In our sample, women were aged between 14 and 45 years and thus they were born between 1960 and 1992. We attempted to deal with this issue by estimating risk ratios for cesarean section according to the SEP-12 among women who are mostly close in age (25–34 years old). We were also concerned about potential confounding due to the number and the mode of previous deliveries being linked to both past socioeconomic context and cesarean rates. Indeed, among women who experienced a surgical delivery, the likelihood of vaginal birth in the current delivery decreases as the number of previous cesarean deliveries increases [57] but this likelihood rises with an increasing number of vaginal births after a cesarean section. [58] Therefore, the number and the mode of previous deliveries could influence the decision to proceed with a cesarean section in the current delivery. In order to address this issue we limited our analysis to women with only one previous delivery. All restricted analysis we performed revealed similar results to those obtained from analyses applied to the whole sample, suggesting that our results were unlikely to have been biased by potential cohort effects or by the number or the mode of previous deliveries.

In summary, this study showed variability in cesarean rates driven by the women´s socioeconomic position at 12 years old, among women with a previous cesarean section. Although the strength of association between these factors is low to moderate, such relationship is not explained by other factors that are usually related with the mode of delivery. While we recognize that a full explanation of the decision-making process concerning mode of delivery requires an understanding of women´s and health professionals’ views on the reasoning behind their options, further research is needed on factors influencing cesarean delivery throughout the life course.

## Supporting Information

S1 Dataset Database including all variables used in statistical analyses here reported. (SAV)

S1 Text Questions on items used to describe the socioeconomic position when the participants were 12 years of age. (DOCX)

## References

[1] World Health Organization Regional Office for Europe. European health for all database (HFA-DB). 2012; Available at: http://data.euro.who.int/hfadb/. Accessed 14 March 2012.

[2] World Health Organization. Appropriate technology for birth. Lancet. 1985; 2: 436–437. 2863457

[3] Liu S, Liston RM, Joseph KS, Heaman M, Sauve R, Kramer MS. Maternal mortality and severe morbidity associated with low-risk planned cesarean delivery versus planned vaginal delivery at term. CMAJ. 2007; 176: 455–460. 17296957 10.1503/cmaj.060870 PMC1800583

[4] Declercq E, Barger M, Cabral HJ, Evans SR, Kotelchuck M, Simon C, et al Maternal outcomes associated with planned primary cesarean births compared with planned vaginal births. Obstet Gynecol. 2007; 109: 669–677. 17329519 10.1097/01.AOG.0000255668.20639.40

[5] Clark EA, Silver RM. Long-term maternal morbidity associated with repeat cesarean delivery. Am J Obstet Gynecol. 2011; 205: S2–S10. 10.1016/j.ajog.2011.09.028 22114995

[6] Henderson J, McCandlish R, Kumiega L, Petrou S. Systematic review of economic aspects of alternative modes of delivery. BJOG. 2001; 108: 149–157. 11236114 10.1111/j.1471-0528.2001.00044.x

[7] PORDATA. Base de Dados Portugal Contemporâneo. 2012. Available at: http://www.pordata.pt/. Accessed 02 February 2012.

[8] Johanson R, Newburn M, Macfarlane A. Has the medicalisation of childbirth gone too far? BMJ. 2002; 324: 892–895. 11950741 10.1136/bmj.324.7342.892 PMC1122835

[9] Simpson KR, Atterbury J. Trends and issues in labor induction in the United States: implications for clinical practice. J Obstet Gynecol Neonatal Nurs. 2003; 32: 767–779. 14649598 10.1177/0884217503258528

[10] Bryant AS, Washington S, Kuppermann M, Cheng YW, Caughey AB. Quality and equality in obstetric care: racial and ethnic differences in caesarean section delivery rates. Paediatr Perinat Epidemiol. 2009; 23: 454–462. 10.1111/j.1365-3016.2009.01059.x 19689496

[11] Cammu H, Martens G, Keirse MJ. Mothers' Level of Education and Childbirth Interventions: A Population-based Study in Flanders, Northern Belgium. Birth. 2011; 38: 191–199. 10.1111/j.1523-536X.2011.00476.x 21884227

[12] Zlot AI, Jackson DJ, Korenbrot C. Association of acculturation with cesarean section among Latinas. Matern Child Health J. 2005; 9: 11–20. 15880970 10.1007/s10995-005-2447-3

[13] Althabe F, Belizan JM, Villar J, Alexander S, Bergel E, Ramos S, et al Mandatory second opinion to reduce rates of unnecessary caesarean sections in Latin America: a cluster randomised controlled trial. Lancet. 2004; 363: 1934–1940. 15194252 10.1016/S0140-6736(04)16406-4

[14] Niino Y. The increasing cesarean rate globally and what we can do about it. Biosci Trends. 2011; 5: 139–150. 10.5582/bst.2011.v5.4.139 21914948

[15] Habiba M, Kaminski M, Da Fre M, Marsal K, Bleker O, Librero J, et al Caesarean section on request: a comparison of obstetricians' attitudes in eight European countries. BJOG. 2006; 113: 647–656. 16709207 10.1111/j.1471-0528.2006.00933.x

[16] Graham WJ, Hundley V, McCheyne AL, Hall MH, Gurney E, Milne J. An investigation of women's involvement in the decision to deliver by caesarean section. BJOG. 1999; 106: 213–220. 10426639 10.1111/j.1471-0528.1999.tb08233.x

[17] Hildingsson I. How much influence do women in Sweden have on caesarean section? A follow-up study of women's preferences in early pregnancy. Midwifery. 2008; 24: 46–54. 17197058 10.1016/j.midw.2006.07.007

[18] Behague DP, Victora CG, Barros FC. Consumer demand for caesarean sections in Brazil: informed decision making, patient choice, or social inequality? A population based birth cohort study linking ethnographic and epidemiological methods. BMJ. 2002; 324: 942–945. 11964338 10.1136/bmj.324.7343.942 PMC102326

[19] Gamble JA. Creedy DK. Women's preference for a cesarean section: incidence and associated factors. Birth. 2001; 28: 101–110. 11380381 10.1046/j.1523-536x.2001.00101.x

[20] Walsh DJ. Childbirth embodiment: problematic aspects of current understandings. Sociol Health Illn. 2010; 32: 486–501. 10.1111/j.1467-9566.2009.01207.x 20003040

[21] Guihard P, Blondel B. Trends in risk factors for caesarean sections in France between 1981 and 1995: lessons for reducing the rates in the future. BJOG. 2001; 108: 48–55. 11213004 10.1111/j.1471-0528.2001.00009.x

[22] Alves B, Sheikh A. Investigating the relationship between affluence and elective caesarean sections. BJOG. 2005; 112: 994–996. 15958007 10.1111/j.1471-0528.2005.00657.x

[23] Leone T, Padmadas SS, Matthews Z. Community factors affecting rising caesarean section rates in developing countries: an analysis of six countries. Soc Sci Med. 2008; 67: 1236–1246. 10.1016/j.socscimed.2008.06.032 18657345

[24] Cesaroni GF, Forastiere F, Perucci CA. Are cesarean deliveries more likely for poorly educated parents? A brief report from Italy. Birth. 2008; 35: 241–244. 10.1111/j.1523-536X.2008.00245.x 18844650

[25] Matijasevich A, Victora CG, Lawlor DA, Golding J, Menezes AM, Araujo CL, et al Association of socioeconomic position with maternal pregnancy and infant health outcomes in birth cohort studies from Brazil and the UK. J Epidemiol Community Health. 2012; 66: 127–135 10.1136/jech.2010.108605 20628081 PMC3245894

[26] Tollanes MC, Thompson JM, Daltveit AK, Irgens LM. Cesarean section and maternal education; secular trends in Norway, 1967–2004. Acta Obstet Gynecol Scand. 2007; 86: 840–848. 17611830 10.1080/00016340701417422

[27] Fairley L, Dundas R, Leyland AH. The influence of both individual and area based socioeconomic status on temporal trends in Caesarean sections in Scotland 1980–2000. BMC Public Health. 2011; 11: 330 10.1186/1471-2458-11-330 21592328 PMC3114726

[28] Witt WP, Wisk LE, Cheng ER, Mandell K, Chatterjee D, Wakeel F, et al Determinants of Cesarean Delivery in the US: A Lifecourse Approach. Matern Child Health J. 2015; 19: 84–93. 10.1007/s10995-014-1498-8 24770955 PMC4209310

[29] Lynch JW, Kaplan GA, Salonen JT. Why do poor people behave poorly? Variation in adult health behaviours and psychosocial characteristics by stages of the socioeconomic lifecourse. Soc Sci Med. 1997; 44: 809–819. 9080564 10.1016/s0277-9536(96)00191-8

[30] van de Mheen H, Stronks K, Looman CW, Mackenbach JP. Does childhood socioeconomic status influence adult health through behavioural factors? Int J Epidemiol. 1998; 27: 431–437. 9698131 10.1093/ije/27.3.431

[31] Lawlor DA, Batty GD, Morton SM, Clark H, Macintyre S, Leon DA. Childhood socioeconomic position, educational attainment, and adult cardiovascular risk factors: the Aberdeen children of the 1950s cohort study. Am J Public Health. 2005; 95: 1245–1251. 15983276 10.2105/AJPH.2004.041129 PMC1449347

[32] Batty GD, Lewars H, Emslie C, Benzeval M, Hunt K. Problem drinking and exceeding guidelines for 'sensible' alcohol consumption in Scottish men: associations with life course socioeconomic disadvantage in a population-based cohort study. BMC Public Health. 2008; 8: 302 10.1186/1471-2458-8-302 18761741 PMC2538536

[33] Jefferis BJ, Power C, Graham H, Manor O. Effects of childhood socioeconomic circumstances on persistent smoking. Am J Public Health. 2004; 94: 279–285 14759943 10.2105/ajph.94.2.279 PMC1448244

[34] Amuzu A, Carson C, Watt HC, Lawlor DA, Ebrahim S. Influence of area and individual lifecourse deprivation on health behaviours: findings from the British Women's Heart and Health Study. Eur J Cardiovasc Prev Rehabil. 2009; 16: 169–173. 10.1097/HJR.0b013e328325d64d 19242356

[35] Graham H, Hawkins SS, Law C. Lifecourse influences on women's smoking before, during and after pregnancy. Soc Sci Med. 2010; 70: 582–587. 10.1016/j.socscimed.2009.10.041 19932931

[36] Hillsdon M, Lawlor DA, Ebrahim S, Morris JN. Physical activity in older women: associations with area deprivation and with socioeconomic position over the life course: observations in the British Women's Heart and Health Study. J Epidemiol Community Health. 2008; 62: 344–350. 10.1136/jech.2006.058610 18339828

[37] Cohen S, Janicki-Deverts D, Chen E, Matthews KA. Childhood socioeconomic status and adult health. Ann N Y Acad Sci. 2010; 1186: 37–55. 10.1111/j.1749-6632.2009.05334.x 20201867

[38] Kuh D, Ben-Shlomo Y, Lynch J, Hallqvist J, Power C. Life course epidemiology. J Epidemiol Community Health. 2003; 57: 778–783. 14573579 10.1136/jech.57.10.778 PMC1732305

[39] Abel T. Cultural capital and social inequality in health. J Epidemiol Community Health. 2008; 62: e13 18572429 10.1136/jech.2007.066159

[40] Teixeira C, Correia S, Victora CG, Barros H. The Brazilian Preference: Cesarean Delivery among Immigrants in Portugal. PLoS One. 2013; 8: e60168 10.1371/journal.pone.0060168 23555912 PMC3608593

[41] Kramer MS, Platt RW, Wen SW, Joseph KS, Allen A, Abrahamowicz M, et al A new and improved population-based Canadian reference for birth weight for gestational age. Pediatrics. 2001; 108: E35 11483845 10.1542/peds.108.2.e35

[42] Severo M, Gaio AR, Lourenco P, Alvelos M, Goncalves A, Lunet N, et al Diagnostic value of patterns of symptoms and signs of heart failure: application of latent class analysis with concomitant variables in a cross-sectional study. BMJ Open. 2012; 2: e001510 10.1136/bmjopen-2012-001510 23148342 PMC3532992

[43] Scharoun-Lee M, Gordon-Larsen P, Adair LS, Popkin BM, Kaufman JS, Suchindran CM. Intergenerational profiles of socioeconomic (dis)advantage and obesity during the transition to adulthood. Demography. 2011; 48: 625–651. 10.1007/s13524-011-0024-5 21491185 PMC3381949

[44] Yuan KH, Bentler PM. Three likelihood-based methods for mean and covariance structure analysis with nonnormal missing data. In: Sobel and Becker, Editors. Sociological Methodology. 2000; 30: 165–200.

[45] Landis JR, Koch GG. The measurement of observer agreement for categorical data. Biometrics. 1977; 33: 159–174. 843571

[46] Barros AJ, Hirakata VN. Alternatives for logistic regression in cross-sectional studies: an empirical comparison of models that directly estimate the prevalence ratio. BMC Med Res Methodol. 2003; 3: 21 14567763 10.1186/1471-2288-3-21 PMC521200

[47] Victora CG, Huttly SR, Fuchs SC, Olinto MT. The role of conceptual frameworks in epidemiological analysis: a hierarchical approach. Int J Epidemiol. 1997; 26: 224–227. 9126524 10.1093/ije/26.1.224

[48] Mazzoni A, Althabe F, Liu N, Bonotti A, Gibbons L, Sanchez A, et al Women's preference for caesarean section: a systematic review and meta-analysis of observational studies. BJOG. 2011; 118: 391–399. 10.1111/j.1471-0528.2010.02793.x 21134103 PMC3312015

[49] Hildingsson I, Radestad I, Rubertsson C, Waldenstrom U. Few women wish to be delivered by caesarean section. BJOG. 2002; 109: 618–623. 12118637

[50] Pang MW, Leung TN, Lau TK, Hang Chung TK. Impact of first childbirth on changes in women's preference for mode of delivery: follow-up of a longitudinal observational study. Birth. 2008; 35: 121–128. 10.1111/j.1523-536X.2008.00225.x 18507583

[51] Wu JM, Viswanathan M, Ivy JS. A Conceptual Framework for Future Research on Mode of Delivery. Matern Child Health J. 2012; 16: 1447–1454 22045022 10.1007/s10995-011-0910-x

[52] Fenwick J, Hauck Y, Downie J, Butt J. The childbirth expectations of a self-selected cohort of Western Australian women. Midwifery. 2005; 21: 23–35. 15740814 10.1016/j.midw.2004.07.001

[53] Krieger N, Davey Smith G. "Bodies count," and body counts: social epidemiology and embodying inequality. Epidemiol Rev. 2004; 26: 92–103. 15234950 10.1093/epirev/mxh009

[54] Galobardes B, Shaw M, Lawlor DA, Lynch JW, Davey Smith G. Indicators of socioeconomic position (part 1). J Epidemiol Community Health. 2006; 60:7–12. 16361448 10.1136/jech.2004.023531 PMC2465546

[55] Hawe P, Shiell A. Social capital and health promotion: a review. Soc Sci Med. 2000; 51: 871–885. 10972431 10.1016/s0277-9536(00)00067-8

[56] Borgonovi F. A life-cycle approach to the analysis of the relationship between social capital and health in Britain. Soc Sci Med. 2010; 71: 1927–1934. 10.1016/j.socscimed.2010.09.018 20943301

[57] Flamm BL. Vaginal birth after caesarean (VBAC). Best Pract Res Clin Obstet Gynaecol. 2001; 15: 81–92. 11359316 10.1053/beog.2000.0150

[58] Mercer BM, Gilbert S, Landon MB, Spong CY, Leveno KJ, Rouse DJ, et al Labor outcomes with increasing number of prior vaginal births after cesarean delivery. Obstet Gynecol. 2008; 111: 285–291. 10.1097/AOG.0b013e31816102b9 18238964

