# Supplementary material for: Socioeconomic Position Early in Adolescence and Mode of Delivery Later in Life: Findings from a Portuguese Birth Cohort
Source: PLoS One. 2015 Mar 23;10(3):e0119517. doi: 10.1371/journal.pone.0119517 (PMC4370463; doi:10.1371/journal.pone.0119517)
Supplement: S1 Text — (DOCX) [file pone.0119517.s002.docx]

**Supporting Information**

S1_Text: Questions on items used to describe the socioeconomic position when the participants were 12 years of age.

What is the education level of your biologic/adopted parents?

Father Mother

No education _____ _____

Primary level of basic school (4 years) _____ _____

Secondary level of basic school (6 years) _____ _____

Tertiary level of basic school (9 years) _____ _____

Secondary school (12 years) _____ _____

BA _____ _____

University degree/graduation _____ _____

Master degree _____ _____

PhD (Doctoral degree) _____ _____

Other. Specify: ______________________________________________________________

I would like that you recall the time when you were 12 years old.

Where did you live?
 Rented house _____
 Owned house _____
 Other. Specify: _____

From the following list of items, which ones did your family hold or had access to?
 Yes No Don’t know

Car ___ ___ ____

Television ___ ___ ____

Bicycle ___ ___ ____

Housemaid ___ ___ ____

Holidays away from home ___ ___ ____

Telephone ___ ___ ____

Washing machine ___ ___ ____

House heating ___ ___ ____

You were member of a social or cultural club or association ___ ___ ____

You were member of a sports club ___ ___ ____
